# Intensification of *Ex Situ* Bioremediation of Soils Polluted with Used Lubricant Oils: A Comparison of Biostimulation and Bioaugmentation with a Special Focus on the Type and Size of the Inoculum

**DOI:** 10.3390/ijerph17114106

**Published:** 2020-06-09

**Authors:** Attila Bodor, Péter Petrovszki, Ágnes Erdeiné Kis, György Erik Vincze, Krisztián Laczi, Naila Bounedjoum, Árpád Szilágyi, Balázs Szalontai, Gábor Feigl, Kornél L. Kovács, Gábor Rákhely, Katalin Perei

**Affiliations:** 1 Department of Biotechnology, University of Szeged, H-6726 Szeged, Hungary; bodora@bio.u-szeged.hu (A.B.); petropet14@gmail.com (P.P.); pennant20@gmail.com (Á.E.K.); vgye@bio.u-szeged.hu (G.E.V.); laczi.krisztian@brc.hu (K.L.); naila@bio.u-szeged.hu (N.B.); szilagyi.arpad@bio.u-szeged.hu (Á.S.); kovacs.kornel@brc.hu (K.L.K.); perei@bio.u-szeged.hu (K.P.); 2 Institute of Environmental and Technological Sciences, University of Szeged, H-6726 Szeged, Hungary; 3 Institute of Biophysics, Biological Research Centre, H-6726 Szeged, Hungary; szalontai.balazs@brc.hu; 4 Doctoral School of Environmental Sciences, University of Szeged, H-6720 Szeged, Hungary; 5 Department of Plant Biology, University of Szeged, H-6726 Szeged, Hungary; feigl@bio.u-szeged.hu; 6 Department of Oral Biology and Experimental Dental Research, University of Szeged, H-6720 Szeged, Hungary

**Keywords:** soil rehabilitation, intensification, TPH biodegradation, used lubricants, inoculation level, *Rhodococcus*

## Abstract

Used lubricant oils (ULOs) strongly bind to soil particles and cause persistent pollution. In this study, soil microcosm experiments were conducted to model the *ex situ* bioremediation of a long term ULO-polluted area. Biostimulation and various inoculation levels of bioaugmentation were applied to determine the efficacy of total petrol hydrocarbon (TPH) removal. ULO-contaminated soil microcosms were monitored for microbial respiration, colony-forming units (CFUs) and TPH bioconversion. Biostimulation with inorganic nutrients was responsible for 22% of ULO removal after 40 days. Bioaugmentation using two hydrocarbon-degrader strains: *Rhodococcus quingshengii* KAG C and *Rhodococcus erythropolis* PR4 at a small inoculum size (10^7^ CFUs g^−1^ soil), reduced initial TPH concentration by 24% and 29%, respectively; the application of a higher inoculum size (10^9^ CFUs g^−1^ soil) led to 41% and 32% bioconversion, respectively. After 20 days, all augmented CFUs decreased to the same level as measured in the biostimulated cases, substantiating the challenge for the newly introduced hydrocarbon-degrading strains to cope with environmental stressors. Our results not only highlight that an increased number of degrader cells does not always correlate with enhanced TPH bioconversion, but they also indicate that biostimulation might be an economical solution to promote ULO biodegradation in long term contaminated soils.

## 1. Introduction

Lubricant oils (LOs) are heavy mineral oil-based products with numerous formulations (5000–10,000) and are used as friction-reducing, cooling and anti-corrosion agents on the mechanical moving parts of machines [1]. Global LO consumption was 35.6 million tons in 2015, of which one third was attributed to North America and Western Europe together (while the Asia-Pacific region itself consumed almost twice that of North America). Up to 56% of outstanding LOs are applied in the internal combustion engines of motorized vehicles such as automobiles, motorcycles and locomotives [2]. During normal operation, LOs undergo a great variety of physicochemical changes accumulating harmful compounds such as combustion products, polychlorinated and polyaromatic hydrocarbons (PCBs and PAHs) as well as heavy metals at a toxic concentration [3]. Therefore, used lubricant oils (ULOs) are considered as the largest liquid hazardous waste stream in Europe [4]. Moreover, 40–50% of the used lubricants end up in the environment as waste via transportation, usage, storage or leaking. In consequence, 5 million tons of ULOs are released just in Europe per year. Once emitted into the environment, ULOs can pose serious risks to human health and natural habitats (both aquatic and terrestrial areas) [3,5,6].

In this regard, soils are extremely compromised, used lubricants can clog pores in soil and strongly bind to soil particles, leading to a persistent pollution [7]. Areas in close proximity to vehicle traffic or maintenance zones are particularly vulnerable, and are considered as high-risk contamination areas [8]. Pollution with oil-derivatives changes the physical, chemical and biological composition of the original soil to such an extent that it might limit the spectra of subsequent uses, therefore the selection of appropriate rehabilitation procedures is crucial.

Bioremediation, which exploits the degradative pathways of microorganisms or plants to neutralize pollutants, is a widespread clean-up technology for petroleum hydrocarbon-contaminated soils either conducted in situ (at the contaminated site) or ex situ (on excavated samples) [9,10,11,12,13,14]. ULOs are less volatile and less biodegradable as compared to other hydrocarbons [15], hence, physicochemical remediation technologies are often preferred, despite the high costs and irreversible damages inflicted to the soil matrices [16,17]. Very little is known about the biodegradation of lubricant oils, the risk of impediment and the complex optimization of these biological processes due to the widely varying characteristics of used lubricants. Yet, the application of bioremediation methods remain the most environmentally friendly and cost-effective rehabilitation technology [8,17,18,19,20]. Biostimulation of native microflora by nutrient addition can accelerate natural biodegradation processes [11,12]. A high level of hydrocarbon pollution as a carbon source can rapidly deplete the concentration of available inorganic nutrients such as nitrogen and phosphorus. Thus, the supplementation with N and P to attain an optimal C/N/P ratio in contaminated soils is preferred to decrease pollution levels. While the ratio of C/N/P = 100/10/(1−5) is often recommended for the mineralization of hydrocarbons in soil, C/N/P = 500/10/1 ratio has proved to be more effective for the biodegradation of waste lubricants [18,21,22]. As another approach to bioremediation, bioaugmentation involves the inoculation of auto- or allochthonous degrader microorganisms into the polluted site. The outcome of bioremediation highly depends on soil conditions and the presence of microbial degrader strains, so in the case of bioaugmentation, the survival of introduced degraders is crucial [9,10,11,12,23]. Despite their higher tolerance to toxicity, augmented cells are often reported to be incapable of coping with environmental stresses or of coexisting with the members of the native microbiome inhabiting contaminated areas [24,25,26,27]. Even if the introduced strains survived on a field scale, their biodegradation performance might be less effective than it was when it had been previously detected under laboratory conditions [28,29]. Although bioaugmentation remains a promising rehabilitation procedure, as a result of the contradictory observations mentioned above, its advantages and capacity have been frequently debated [13,30,31,32,33,34,35].

According to U.S. EPA guidelines, bioremediation can be accomplished in soils even with 10^3^ colony forming units (CFUs) g^−1^ [36]. Regarding bioaugmentation, the key point is to maintain a high microbial biomass having a major impact both on the efficacy and the cost of the rehabilitation procedure [37,38]. Nevertheless, standard recommendations for the level of inoculation are lacking. In practice, inoculum sizes applied in soils vary widely, including but not limited to 10^5^ CFU g^−1^ [36], 5 × 10^5^ CFU g^−1^ [39], 10^7^ CFU g^−1^ [24,25], 5 × 10^7^ CFU g^−1^ [31], 10^7^–10^8^ CFU g^−1^ [40], 10^8^ CFU g^−1^ [32,34,41,42] and 2 × 10^8^ CFU g^−1^ [30]. It is often considered that the higher the level of inoculation, the more effective decontamination can be achieved. Preparation of an increased inoculum size, however, can significantly increase the costs of bioaugmentation. In contrast to a large inoculum fraction, acclimation and growth of exogenous bacteria can be retarded or inhibited when introduced into polluted soils with a smaller inoculum size [37]. Despite the extensive amount of studies published on hydrocarbon biodegradation, our knowledge on the bioremediation of ULO-polluted soils and its affecting factors is yet limited.

In this study, Fourier transform infrared spectroscopy (FTIR) was used to elucidate changes in the chemical composition of LOs, and their derivatives exposed to natural weathering processes in soil in order to explore the biodegradative potential of the indigenous microbial community. Both commercially available and newly isolated bacterial strains were tested for LO biodegradation prior to constructing soil microcosm experiments. Ex situ bioremediation of ULO-polluted soil was modelled through biostimulation and bioaugmentation approaches. Changes in microbial respiration activity, viable cell counts and total petrol hydrocarbon (TPH) bioconversion were followed to compare the feasibility of biostimulation and bioaugmentation as potential treatments in a local, long term ULO-polluted railway station area. Bioaugmentation studies were conducted at a low (approximately 10^7^ CFU g^−1^) and a high level (approximately 10^9^ CFU g^−1^) of inoculation in order to assess the effects of applied inoculum size on ULO biodegradation in soil. The obtained results may not only contribute to broaden our knowledge on the rehabilitation of long term ULO-polluted soils but may also bring a new insight into the bioremediation processes applicable in soils polluted by used lubricants.

## 2. Materials and Methods 

### 2.1. Chemicals

Fresh MK8 lubricant oil was provided by MÁV Hungarian State Railways (Hungary). All chemicals and analytical grade solvents were obtained from standard commercial suppliers (Reanal, Sigma-Merck, VWR International).

### 2.2. Soil Sampling and Physicochemical Characterization

The soil samples used in our experiments originated from a railway marshalling yard near Szeged (Hungary), where ULOs, leaking from locomotives and polluting the soil, have been a long-standing environmental problem.

**Figure 1:**
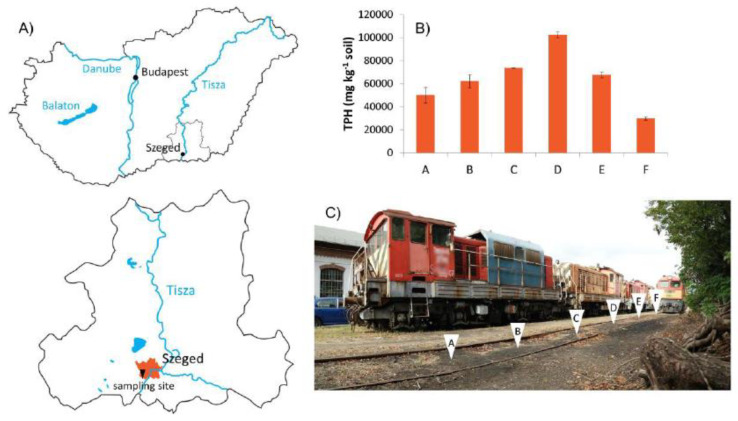
(**A**) Location of the used lubricant oil (ULO)-contaminated sampling site near Szeged, Hungary and (**B**) total petrol hydrocarbon (TPH) levels measured in (**C**) six sampling points of the transect.

ULO-polluted average soil samples (approximately 5000 g each) were collected at six different sampling points (Figure 1) from the topsoil (0–25 cm), along a transect within approximately 2 m distance of each other. An uncontaminated control sample was also taken from an area directly adjacent to the polluted site, representing a soil condition before extreme anthropogenic effects of oil spills. Vertisols are the prevailing soil types in this area according to FAO classification [43]. Stones, gravels and plant debris were removed by hand from the air-dried and homogenized soils. Prior to air-drying, field moisture was determined gravimetrically by overnight oven-drying at 105 °C [31]. ULO-polluted soils from the six sampling points were mixed in equal proportions, then once again thoroughly homogenized to form a composite. Soil samples were stored in open plastic bags at 4 °C in the dark until required.

Soil samples were analyzed for several physicochemical characteristics: moisture content, soil texture, pH and electrical conductivity (EC) were determined according to Gangwar et al. [44]. In brief, pH was measured in the mixture of soil:distilled water = 1:2.5, and EC was determined in a soil paste completely saturated with water. Saturation percentage (SP) was expressed as the water content of this saturated soil paste [45]. Soil salinity was calculated using the values of EC and SP according to Stefanovits et al. [46]. The modified method of Naeth et al. [47] was used for the determination of the water holding capacity (WHC). Shortly, 35 g soil was weighed into a plastic cylinder (the bottom of which was secured with cotton fabric and a rubber band), saturated for 48 h and then placed onto damp sand to drain for 48 h. Soil organic matter was indicated by the weight loss on ignition at 550 °C for 4 h, while carbonate content in soil was measured by combustion at 950 °C for 2 h [48]. Total C and total N in soils were analyzed using a Vario MAX CN Analyzer equipment (Elementar Group, Hanau, Germany) with the operating parameters described in Kakuk et al. [49]. For available P determination, the optimized colorimetric method of Franson [50] was used. In brief, 2.5 g soil (moist weight) was suspended in 5 mL distilled water and shaken for 30 min at 150 rpm. After centrifugation (13,000 rpm, 5 min), 900 µL of the supernatant was mixed with 256 µL Vanadate-molybdate reagent (VM: 1.25 g L^−1^ NH_4_VO_3_, 25 g L^−1^ (NH_4_)_6_Mo_7_O_24_ x 4H_2_O and 330 mL L^−1^ cc. HCl), 128 µL distilled water and 10 µL cc. sulfuric acid, followed by an incubation step of 10 min. Absorbance values measured at 405 nm were used to calculate phosphate concentration from the calibration curve.

### 2.3. Chemical Characterization of Lubricants

Chemical properties of fresh MK8 lubricant oil and spent ULOs extracted from polluted soils were analyzed with Fourier transform infrared spectroscopy (FTIR) using a Bruker Wertex70 spectrometer (Bruker Corp., Billerica, Germany) equipped with a Platinum Diamond single-reflection attenuated total reflection (ATR) unit and a liquid nitrogen-cooled mercury cadmium telluric (MCT) detector [51]. Carbon disulfide was used as a solvent. A drop of extract was analyzed on a diamond crystal by scanning the 4000–900 cm^−1^ range. Next, 128 co-added interferograms with 2 cm^−1^ spectral resolution were used to calculate the infrared spectra of LOs. Diamond crystal was washed twice with the mixture of methanol and chloroform (1:1, v v^−1^) prior to each measurement and a reference single-beam spectrum was recorded to check the cleanness of the diamond. Absorption spectra were calculated by comparing the single-beam spectra of LOs to those of the single-beam reference spectra of the diamond.

### 2.4. Bacterial Strains

*Rhodococcus erythropolis* PR4 (NBRC 100887) was obtained from National Institute of Technology and Evaluation, Biological Resource Center (NBRC, Kisarazu-shi, Chiba, Japan). *R. erythropolis* is a well-characterized marine bacterium able to degrade various hydrocarbons [52,53,54].

Strain KAG C was newly isolated from diesel oil, which was stored in a fuel tank of a machine repair site, owned by ‘Új Élet’ Agricultural Production Co-operative (Székkutas, Hungary). Morphological, physiological and biochemical characterization of the new isolates were performed following the methods developed by Cowan et al. [55]. Bacterial genomic DNA of strain KAG C was isolated with GenEluteTM Bacterial Genomic DNA Kit (Merck KGaA, Darmstadt, Germany), following the manufacturer’s instructions. PCR amplification of the 16S rDNA gene was performed using Phusion^®^ High Fidelity DNA Polymerase (Thermo Fisher Scientific Inc., Waltham, USA) and primers 27f (5’-AGAGTTTGATCMTGGCTCAG-3’) and 1492r (5’-TACGGYTACCTTGTTACGACTT-3’) with the following conditions: pre-denaturation at 94 °C for 2 min, then 30 cycles of denaturation at 94 °C for 45 sec, annealing at 55 °C for 30 sec, elongation at 72 °C for 1 min. At the end, the reaction mixture was kept at 72 °C for 5 min and then cooled to 4 °C. The PCR product was separated by agarose gel electrophoresis and purified with GeneJET Gel Extraction Kit (Thermo Fisher Scientific Inc., Waltham, USA). Sequencing was performed by Eurofins Genomics (Germany) and 16S rDNA sequence of strain KAG C was determined and compared to the analogous sequences of bacterial strains in the NCBI GenBank database [56].

Both strains were maintained on Luria Bertani (LB) broth and agar plates and stored at 4 °C [54].

### 2.5. Lubricant Oil Biodegradation Tests

Starter cultures and inocula were prepared according to Laczi et al. [54]. Cells were inoculated in 1% (v v^−1^) into 160 mL serum vials (Merck KGaA, Germany) containing fresh minimal medium (MM: 0.68 g L^−1^ KH_2_PO_4_, 0.87 g L^−1^ K_2_HPO_4_, 0.58 g L^−1^ NaCl, 0.125 g L^−1^ MgSO_4_ × 7H_2_O, 0.044 g L^−1^ CaCl_2_ × 2H_2_O, 1.2 g L^−1^ NH_4_NO_3_, 0.014 g L^−1^ FeSO_4_, 0.0093 g L^−1^ EDTA, 0.0002 g L^−1^ ZnSO_4_ × 7H_2_O, 0.00006 g L^−1^ MnCl_2_ × 7H_2_O, 0.0006 g L^−1^ H_3_BO_4_, 0.0004 g L^−1^ CoCl_2_ × 6H_2_O, 0.00002 g L^−1^ CuCl_2_ × 2H_2_O, 0.00004 g L^−1^ NiCl_2_ × 6H_2_O and 0.000046 g L^−1^ NaMoO_4_ × 6H_2_O) [53] supplemented with 1% (m v^−1^) fresh MK8 lubricant oil at a final volume of 20 mL aqueous phase and a 140 mL headspace. Cell-free samples were used as controls. All vials were capped with butyl rubber septa, then incubated in a rotary shaker (160 rpm at 28 °C) for 16 days. Since MK8 lubricant oil was used as the sole carbon source in each vial, increasing the CO_2_ level was considered as an indirect measure for LO biodegradation. Microbial respiration was measured with gas chromatography as described below. At the end of the experiment, remaining LO was extracted using 10 mL diethyl ether and measured gravimetrically [57].

### 2.6. Preparation of Soil Microcosms

Soil microcosms were constructed to study the effectiveness of various supplementations, as well as various bacterial inoculum sizes for ULO-polluted soil clean-up. Experimental setups for each type of soil microcosms are presented in Table 1.

Proportions of 3 g from polluted composite soil (based on dry soil weight) were placed in butyl rubber capped serum vials (65 mL; Merck KGaA, Germany). Soil samples were not autoclaved. A C/N ratio of nearly 500/10 was considered close to ideal for the microbial degradation of waste lubricant oils [18]. Thus, depending on treatment types, sterile water or liquid mineral medium were supplemented to attain 30% soil moisture (approximately 60% of WHC) [41,58], except for non-treated contaminated controls. For bioaugmentation treatments, starter cultures were prepared according to Laczi et al. [54]. Afterwards, the cells were resuspended in liquid minimal medium to provide 30% soil moisture. ULO-polluted soils were combined with bacterial cells to reach approximately 10^7^ cells g^−1^ for the small inoculum size [24,25,31] and approximately 10^9^–10^10^ cells g^−1^ in cases of the increased inoculum size samples [40]. Vials were opened under sterile conditions and their headspaces were refreshed every two days to achieve sufficient aeration, then sealed again for further microbial respiration monitoring. Each vial was incubated in the dark at 28 °C for 40 days.

### 2.7. Microbial Respiration Monitoring

Microbial respiration activity (RA) was monitored using gas chromatography. For CO_2_ analysis, a gas sample (25 μL) from headspace was manually injected into a Shimadzu GC-2010 gas chromatograph (Shimadzu Corp., Kyoto, Japan) mounted with a thermal conductivity detector (TCD) and an HP PLOT-Q column (30 m × 0.32 mm × 240 μm). Nitrogen was used as a carrier gas. Inlet pressure was set to 70.9 kPa and the split ratio was 4:1 with a colum flow rate of 1.25 mL min^−1^. Temperature of the oven, inlet and TCD were 200 °C, 80 °C and 160 °C, respectively. Analyses were previously calibrated. Respiration activities in LO biodegradation tests were expressed as relative air% of CO_2_. Results from microcosm studies were determined on a cumulative basis of mg of CO_2_ g^−1^ soil or on the amount of CO_2_ produced within 48 h normalized to 1 g soil: mg CO_2_ g^−1^ soil 48 h^−1^.

### 2.8. Enumeration of Aerobic Heterotrophic Bacteria in Soil Microcosms

The number of total cultivable aerobic heterotrophic bacteria (AHB) was determined using the modified method of Wu et al. [32]. Homogenized soil samples (0.15 g soil from each vial) were suspended in 1 mL of 0.9% (m v^−1^) sterile saline solution and vigorously mixed with vortex for 2 min. Subsequent serial dilutions of each soil suspension were plated on LB agar plates. Colony-forming units of AHB were enumerated after three days of incubation at room temperature. Results were expressed as logCFU g^−1^ of dry soil weight.

### 2.9. Determination of Hydrocarbon Bioconversion in Soil Microcosms

After 40 days of incubation, a modified method of Tsuboi et al. [59] was used to quantify residual TPH in soil microcosms. Briefly, 0.5 g soil sample (previously homogenized and dried at 105 °C) was extracted with 5 mL of carbon disulfide [60], shaken vigorously for 1 h and centrifuged (13,000 rpm, 3 min) to separate soil particles from the liquid phase. The supernatant was collected into a fresh tube, then analyzed with an infrared oil-measurement instrument Infracal TOG/TPH Analyzer CVH-1 (Wilks Enterprise Inc., Norwalk, USA). Bioconversion yields of ULOs were determined by the following Equation [53]:

Bioconversion (%) = [(TPH_in non-treated soil_ − TPH_in treated soil_)/TPH_in non-treated soil_] × 100, using the calibration curves to determine TPH values of the samples.

### 2.10. Statistical Analysis

Data sets generated in this study are expressed as mean values with their standard errors. Statistical analyses were performed with SigmaPlot 11.0 software (Systat Software Inc., Erkeath, Germany) using one-way analysis of variance (ANOVA) followed by Duncan’s multiple range test (DMRT) at 5% significance level. 

## 3. Results and Discussion

### 3.1. Soil Characteristics

#### 3.1.1. Uncontaminated Soil

Agronomic parameters of the uncontaminated soil sample are presented in Table 2. The uncontaminated sample was taken from close vicinity to the ULO-polluted site and used as a control representing the initial conditions prior to oil spills. This soil was darkly colored, probably due to the relatively high level of soil organic matter (23.5%). pH measurements estimated a near-neutral pH (pH = 7.79), optimal for microbial activity. Uncontaminated soil texture was classified as clay, which is in line with its high water holding capacity (47.2%). Fine clay grains strongly adsorb water, thus hampering microorganisms’ and plants’ accession to it. Therefore, biological processes are decelerated, even though a high amount of water is stored in clay soils [61]. Hydrocarbons can either be bound to soil organic matter or clay particles, reducing their bioavailability [62]. Clay soils are also usually characterized by a low porosity, which provides less space for bacterial growth and insufficient aeration, which are unfavorable conditions for hydrocarbon biodegradation [63]. Based on the slight salinity (0.11%) and genetic soil type maps [64], the uncontaminated soil might be classified as a Solonchak-like meadow soil. These soils are usually distinguished by a dark color and a high concentration of soil organic matter. The upper soil layer can be salt-affected by salt accumulation which might be due to extreme fluctuations in soil moisture [43,46]. This can be an inhibiting factor for salt-sensitive plant species or microorganisms.

#### 3.1.2. Used Lubricant Oil-Contaminated Soil

TPH concentrations measured in the 6 ULO-contaminated soil samples varied from 29,600 mg kg^−1^ to 102,100 mg kg^−1^ (Figure 1). Main parameters of the composite soil consisting of these 6 ULO-contaminated soil samples, mixed in equal proportion, are given in Table 3.

Although a considerable level of hydrocarbon pollution (64,100 mg TPH kg^−1^ soil) was measured in the composite soil, cell counts of aerobic heterotrophic bacteria were only slightly lower as compared to CFUs in non-polluted soil. The organic matter content of ULO-polluted soil was similar to the non-contaminated sample and not like the C/N ratio, which was considerably higher in the polluted soil (C/N = 47.7) as compared to the unspoiled control (C/N = 34.5). Considering the high TPH content, a depletion of total N presumably occurred due to *in situ* hydrocarbon biodegradation processes prior to the sampling. Available P was detected at a low concentration (32 mg kg^−1^). The result is consistent with the nutrition household of Solonchak-like meadow soils, which are often proved to be extremely changing. Mobility and uptake of P might be retarded by iron compounds in this soil type. Additionally, the release of N compounds is likewise slow and N is often adsorbed to amorphous soil colloids. Thus, even a rich nutrient pool can be hardly accessible for plants and bacteria [46]. The ratio of C/N/P = 477/10/0.089 determined in the ULO-contaminated composite soil significantly differed from the recommended ratio of C/N/P = 100/10/(1-5) for hydrocarbon biodegradation [21,22]. Nevertheless, the C/N ratio of 477/10 itself was close to 500/10, previously reported to be efficient for the microbial degradation of waste LOs according to Lee et al. [18]. Considering the physicochemical profile of the ULO-polluted soil, which revealed poorly available P, we assumed that supplementation of inorganic nutrients could accelerate the biodegradation process of ULOs by the native microbial population.

### 3.2. Lubricant Oil Characteristics

**Figure 2:**
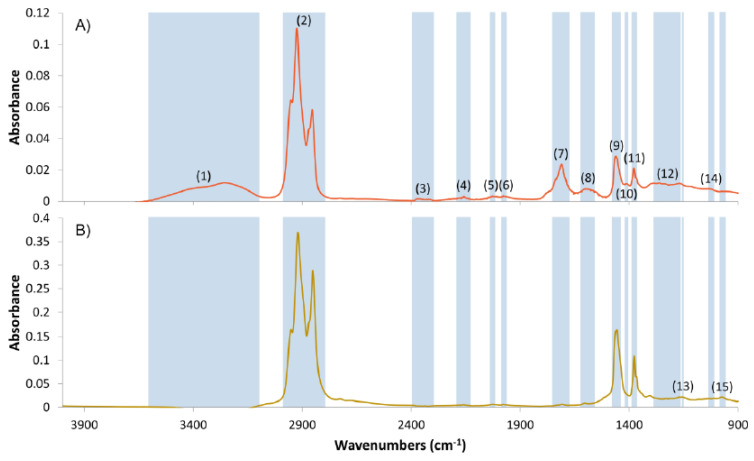
Fourier transform infrared spectroscopy (FTIR) spectra of MK8 lubricant oil: (**A**) spent ULO and (**B**) fresh LO. Absorbance bands: (1) O-H stretching in alcohols; (2) C-H stretching in hydrocarbons; (3) NH_2_^+^ deformation and NH^+^ stretching in amines; (4) Si-H stretching; (5) N=C=S stretching in isothiocyanates; (6) C-H bending in aromatics; (7) C=O stretching in esters, ketones and carboxylic acids; (8) C-C stretching in aromatic rings; (9) C-H bending in hydrocarbons; (10) S=O stretching in sulfates and sulfonates; (11) C-H branching vibration in hydrocarbons; (12) C-O-C stretching in esters and ethers; (13) sulfonate salts, methacrylates; (14) C-N stretching in amines; (15) P-O-C and P=S bonds in zinc dialkyl dithiophosphates (ZDDPs).

Based on the comparison of FTIR spectra of spent ULO and fresh MK8 LO (Figure 2), the main components detected in LOs were open-chain and branched paraffins [65,66] according to the absorbance bands of C-H stretching vibrations (2952–2851 cm^−1^), CH_2_ and CH_3_ angular deformation at around 1460 cm^−1^ and 1376 cm^−1^, respectively [67]. Absorbance bands of C-H stretching vibrations were slightly shifted in spent ULO compared to fresh MK8 LO possibly due to *in situ* ULO-degradation by naturally occurring microorganisms in the polluted soil.

Alcohols and carboxylic acids, which are intermediates in the aerobic degradative pathways of hydrocarbons, were also present in spent ULOs according to the absorbance bands of O-H (3550–3200 cm^−1^) and C=O stretching vibrations (1709 cm^−1^), respectively [1,67,68]. Bands corresponding to C-O-C stretching in esters (1747–1701 cm^−1^) or NH_2_^+^ deformation and NH^+^ stretching in amines (2371 cm^−1^) [65,69] further proved the presence of metabolically active microbes in the polluted soils despite the high level of ULO-contamination. Metal-containing additives of LOs (zinc dialkyl dithiophosphates, ZDDPs), detergents (sulfonates, phenolates and carboxylates) and antifoams were also identified based on the absorption bands of their P-O-C (1050-920 cm^−1^), P = S (1050–920 cm^−1^) and Si-H (2160–2120 cm^−1^) vibrations [69,70,71]. An increased concentration of aromatics (1600 cm^−1^) was also detected in spent ULO compared to the FTIR spectrum of fresh LO and was considered as a result of the normal usage of lubricants [69].

### 3.3. Strain Characterization and Identification

*Rhodococcus erythropolis* PR4 and strain KAG C were applied for the bioaugmentation of ULO-polluted soil. According to our results, strain KAG C is a Gram-positive, aerobic bacterium with oxidase-, catalase-, urease-, weak lipase- and nitrate reductase activities. Colonies formed on LB plates are circular shaped with undulate margins, cream-colored, opaque, flat with a rough surface and brittle texture. Growing on liquid LB, the cells formed aggregates, making accurate cell counting difficult. The strain has an optimal growth in the temperature range of 25–28 °C. Cell growth was observed below 3.5% (m v^−1^) salt concentration. According to the comparative analysis of 16S rDNA gene sequence, strain KAG C was identified as *Rhodococcus quingshengii* (99.5% identity).

### 3.4. Soil Microcosm Experiments

#### 3.4.1. Microbial Respiration and Cell Counts

**Figure 3:**
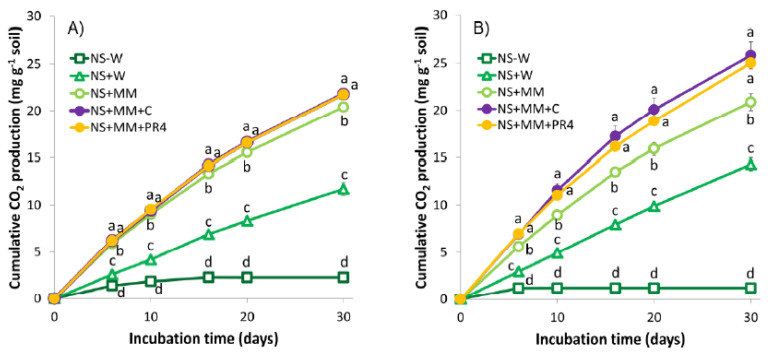
Cumulative CO_2_ production in soil microcosms over 30 days, when applying a (**A**) smaller and a (**B**) larger size of inoculum for bioaugmentation. Different letters in the same remediation time indicate statistical differences among treatments (*n* = 3, *p* ≤ 0.05).

**Figure 4:**
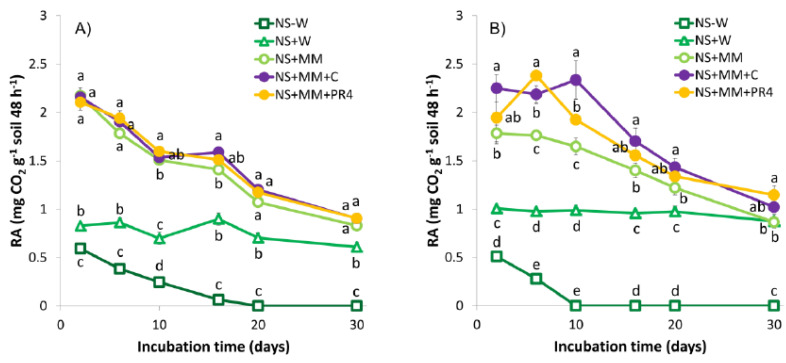
Respiration activity (RA) in soil microcosms over 30 days, when applying a (**A**) smaller and a (**B**) larger size of inoculum for bioaugmentation. Different letters in the same remediation time indicate statistical differences among treatments (*n* = 3, *p* ≤ 0.05).

Based on their respiration activities demonstrated by CO_2_ evolution, microbial communities were viable and active in all of the soil microcosms (Figure 3 and Figure 4).

**Figure 5:**
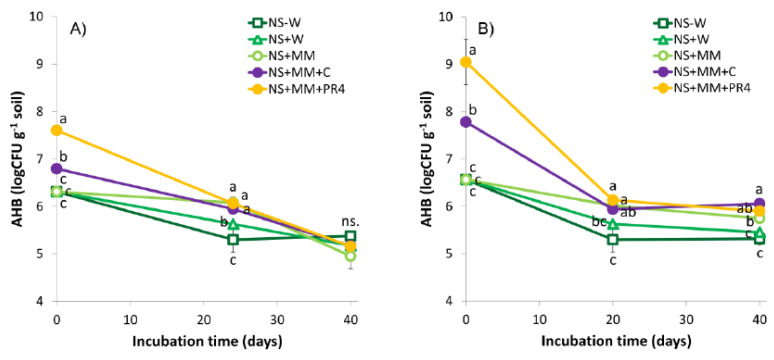
Changes in the numbers of aerobic heterotrophic bacteria (AHB) in soil microcosms when applying a (**A**) smaller and a (**B**) larger size of inoculum for bioaugmentation. For comparison, the CFUs of the non-treated, moistured and biostimulated samples are also displayed. Different letters in the same remediation time indicate statistical differences among treatments (*n* = 3, *p* ≤ 0.05).

**Figure 6:**
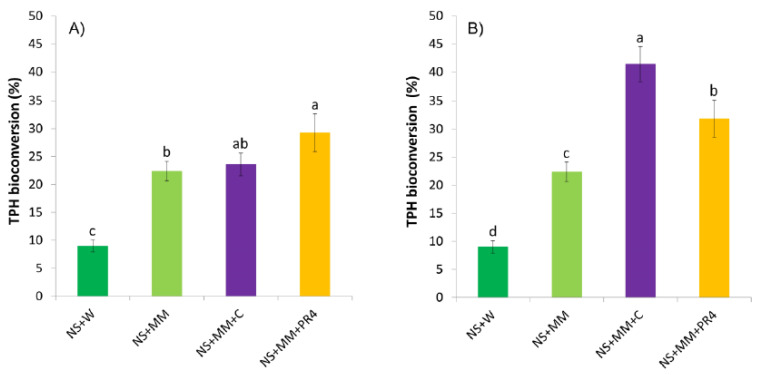
Bioconversion of total petrol hydrocarbons (TPH) in soil microcosms after 40 days, when applying a (**A**) smaller and a (**B**) larger size of inoculum for bioaugmentation. Different letters indicate statistical differences among treatments (*n* = 15, *p* ≤ 0.05).

Even non-treated control soils (NS-W) exhibited a low respiration rate during the first 10–20 days of the incubation due to the original soil water content. Adequate soil moisture is a crucial parameter for biodegradation of soil contaminants. According to Pramer and Bartha [72], the optimal activity of the aerobic soil microbiota requires a water content about 50–70% of WHC. Below that value, insufficient water availability and, above that, limited oxygen supply can lead to inhibitory effects on microbial processes [73]. NS + W samples were amended with water to attain 30% soil moisture (approximately 60% of WHC) in order to simulate natural attenuation. These samples exhibited a steady respiration activity throughout the whole experiment (Figure 4). Soil samples stimulated by liquid mineral medium supplementation (NS + MM) produced more CO_2_ (Figure 3 and Figure 4) due to the input of inorganic nutrients (N, P), adjusting the ratio of C/N to the optimal for TPH removal and providing available P [18,21]. Bioaugmentation approaches, including the inoculation of *R. quingshengii* KAG C (NS + MM + C) and *R. erythropolis* PR4 (NS + MM + PR4) using a small (Figure 3A and Figure 4A) and a large (Figure 3B and Figure 4B) inoculum size, resulted in an enhanced respiration activity. Both strains were able to degrade fresh LO, according to their respiration activities (Figure S1) and TPH bioconversions (Figure S2) in preliminary biodegradation tests. The soil samples used in this study contained a relatively high level of organic matters, thus, CO_2_ evolution itself cannot be considered exclusively as a result of hydrocarbon removal. Nevertheless, enhanced mineralization of easily biodegradable ULO components by the increased initial biomass (Figure 5B) could be responsible for the initially higher level of CO_2_ production in large inoculum sized NS + MM + C and NS + MM + PR4 (Figure 4B) samples. This claim was supported by the TPH bioconversion results (Figure 6B).

ULO-contaminated composite soil contained on average 2.04 × 10^6^ cultivable aerobic heterotrophic bacteria per gram. CFUs in NS−W, NS + W and NS + MM samples decreased slightly throughout the incubation period, potentially due to the minor loss of initial soil moisture. Bioaugmentation strategies involved an increased initial cell count by nearly one and three orders of magnitude in case of the application of a smaller and larger inoculum size (Figure 5), respectively. This higher initial biomass of *R. quingshengii* KAG C and *R. erythropolis* PR4 (when a larger inocula was applied (Figure 5B)) resulted in an enhanced initial CO_2_ evolution in bioaugmented samples (Figure 4B). CFUs counted in all inoculated samples, including both inoculum sizes, decreased considerably during the first 20 days (Figure 5). At the end of the experiment, their cultivable AHB numbers dropped and were comparable to the non-treated, naturally attenuated and biostimulated soil samples.

Several explanations might exist for the cell loss in the bioaugmented samples. Bioremediation of an aged contaminated soil can be difficult due to the strong adsorption of hydrocarbons to soil components (e.g., organic matter, soil matrix, clay particles and minerals). In addition, the accumulation of toxic intermediates and metabolites can take place. These soil characteristics may result in decreased bioavailability of contaminants or repressed microbial activity [31,32,34,62]. Alternatively, the boost of hydrocarbonoclastic bacteria can cause a quick depletion of easily accessible and degradable hydrocarbons in the early stage of bioremediation. As a result, the remaining fractions of hydrocarbon pollutants are often more recalcitrant and less bioavailable, thus inhibiting the proliferation of microorganisms and resulting in a reduced microbial biomass [22,36,62]. It has been also noted that introduced strains might not be able to coexist with the indigenous micro-community members [30,33,74] or cope with multiple environmental stressors and therefore, enter a zero- or low-activity state [26,75,76].

#### 3.4.2. Bioconversion of Hydrocarbons

The soil microcosm tests lasted for 40 days and were performed to assess the biodegradative potential of the native microbial community and to compare the efficiency of small and large bioaugmentation. ULO bioconversion yields, calculated from the remaining TPHs in soil samples, are presented in Figure 6. After 40 days of incubation, poor ULO removal (9%) was observed in NS + W samples (30% soil moisture is approximately equivalent to 60% of WHC in this case). Similar results have been reported by Lee and colleagues [77], who measured 28% heavy mineral oil elimination after 105 days of incubation in soil with water levels of 50% and 80% of WHC. An adequate water supply in the soil is essential for the microbial activity [73], however, high levels of soil moisture can have inhibitory effects on heavy mineral oil biodegradation [77]. In the present case, ULO bioconversion in biostimulated NS + MM sample reached 22% after 40 days (Figure 6), which is consistent with the 105 days waste lubricant decontamination percentage of 50% reached by Lee and colleagues [18]. TPH bioconversions in NS + W and NS + MM not only provide evidence for the presence of adapted microbial strains capable of ULO biodegradation in heavily oil-polluted soils [78], but further proves that the adjustment of the soil C/N ratio to 500/10 and supplementation of nutrients can effectively stimulate the microbial activity, and thus, the biodegradation of lubricants by the indigenous microbial community [18].

In the case of the application of a relatively small-sized inoculum, TPH removals in the NS + MM + C and NS + MM + PR4 samples were 24% and 29%, respectively (Figure 6A). According to the statistical analysis, bioaugmentation with *Rhodococcus quingshengii* KAG C did not significantly improve the biodegradation performance as compared to the simple nutrient supply (NS + MM). Meanwhile, the inoculation of *Rhodococcus erythropolis* PR4 proved to be slightly more effective. These results correlate with previous studies discussing the efficacy of bioaugmentation approaches. Although Roy and colleagues [39] demonstrated that bioaugmentation combined with biostimulation effectively accelerated the bioremediation of a petroleum refinery sludge even with the application of 5 × 10^5^ CFU g^−1^, bioaugmentation techniques often underperform compared to natural attenuation or biostimulation [13,31,34,38]. This may be particularly relevant for the bioremediation of such an aged contaminated soil, where the native soil microbiota might have been already adapted to the presence of hydrocarbon contamination and where exogenous degrader strains do not necessarily survive or perform effectively [26,75,79].

The application of a larger inoculum size of *R. quingshengii* KAG C significantly increased the ULO biodegradation, however, no significant changes were observed when samples were bioaugmented with a large-sized inoculum of *R. erythropolis* PR4 (Figure 6B). After 40 days of incubation, TPH bioconversions of 41% and 32% were achieved in NS + MM + C and NS + MM + PR4, respectively. These findings not only indicate that a successful bioaugmentation treatment is strongly dependent on the degrader strain to be applied and its inoculum size, but further corroborates the idea that after a certain level of inoculation, an increased number of degrader cells used for bioaugmentation does not necessarily result in an enhanced bioconversion of soil pollutants. This phenomenon was previously reported only in aqueous systems [80,81]. According to the best of our knowledge, this study represents the first attempt to examine the effects of inoculum size on the bioaugmentation treatment of a long term ULO-contaminated soil. Our results again support previous observations [30], where it was found that bioaugmentation can be a quick but not always an adequate approach to remove hydrocarbon contaminants from urban soil.

Taking both the considerable loss in CFUs during the incubation (Figure 5B) and the slight increase in ULO bioconversion as a result of large-sized inoculum into account (Figure 6B), application of a smaller inoculum size or stimulating the autochthonous microbiota could be economically more suited and justified treatments for a scaled-up bioremediation study in the future.

## 4. Conclusions

The findings of this study further support the feasibility of biostimulation and bioaugmentation as treatment options for a local, long term ULO-polluted soil. All bioremediation strategies increased the microbial activity in soil, although by the end of the experiments, the cultivable cell counts of AHB significantly decreased in inoculated soils, possibly due to multiple or a combination of limiting factors, such as the declining bioaccessibility of contaminants, an accumulation of toxic intermediates and the transition of introduced strains into a viable but non-culturable (VBNC) state induced by environmental stresses or even cell death. TPH bioconversions obtained with bioaugmentation approaches proved to be affected by the size of the inoculum. Applying a smaller inoculum size of the newly isolated hydrocarbon-degrader *Rhodococcus quingshengii* KAG C did not improve the biodegradation performance compared to the biostimulation treatment, while the low level of inoculation with *Rhodococcus erythropolis* PR4 proved to be more effective. By contrast, the use of a larger size of the *R. quingshengii* KAG C inoculum led to a significantly enhanced ULO biodegradation. Meanwhile, the application of a larger inoculum size did not cause a significantly enhanced performance in ULO bioconversion by *R. erythropolis* PR4 compared to the use of the smaller size of the inoculum. These findings substantiate that, although the success of bioaugmentation strongly depends on the bacterial strain to be applied and its cell density, increasing the number of degrader cells does not necessarily have a positive correlation with increased soil decontamination. Therefore, the application of a smaller inoculum size for allochthonous bioaugmentation or the biostimulation of the autochthonous microbiota is likely more economically justified in order to enhance ULO biodegradation in long term polluted soils. Nevertheless, when an urgent remediation is required, bioaugmentation with carefully selected strains might significantly increase the efficiency of the rehabilitation processes.

## Figures and Tables

**Table 1:** Microcosm experimental setup.

Soil Microcosm	Amendment	Condition
NS ^1^ − W	None (no treatment, no water amendment)	Non-treated control
NS + W	Amended with water (30% soil moisture)	Natural attenuation
NS + MM	Amended with liquid minimal medium (30% soil moisture)	Biostimulated
NS + MM + C	Same as NS+MM plus inoculated with *Rhodococcus quingsenghii* KAG C (approximately 10^7^ cells g^−1^ soil when applying a smaller inoculum size or approximately 10^9^ cells g^−1^ soil when applying a larger inoculum size)	Biostimulated and bioaugmented (KAG C)
NS + MM + PR4	Same as NS+MM plus inoculated with *Rhodococcus erythropolis* PR4 (approximately 10^7^ cells g^−1^ soil when applying a smaller inoculum size or approximately 10^9^ cells g^−1^ soil when applying a larger inoculum size)	Biostimulated and bioaugmented (PR4)

^1^ NS: non-sterilized, ULO-polluted composite soil.

**Table 2:** Agronomic parameters of the uncontaminated soil.

Main Characteristics	Values
pH	7.79 ± 0.02
EC ^1^ (mS cm^−1^)	2.18 ± 0.04
SP ^2^ (%)	61.6 ± 0.3
WHC ^3^ (%)	47.2 ± 0.7
Field moisture (%)	17.9 ± 0.2
Texture	Clay soil
Salinity (%)	0.11 ± 0.00
Carbonates (%)	1.9 ± 0.5
C/N ratio	34.5 ± 2.0
LOI_550_^4^ (%)	23.5 ± 0.5
AHB ^5^ (logCFU g^−1^)	6.78 ± 0.06

^1^ EC: electric conductivity; ^2^ SP: saturation percentage; ^3^ WHC: water holding capacity; ^4^ LOI_550_: loss on ignition at 550 °C; ^5^ AHB: aerobic heterotrophic bacteria.

**Table 3:** Main parameters of the ULO-contaminated composite soil.

Main Characteristics	Values
Total carbon (g kg^−1^)	172.23 ± 4.86
Total nitrogen (mg kg^−1^)	3610 ± 90
Available phosphorus (mg kg^−1^)	32 ± 4
C/N ratio	47.7 ± 0.9
TPH ^1^ (mg kg^−1^)	64100 ± 9900
LOI_550_^2^ (%)	21.7 ± 0.3
AHB ^3^ (logCFU g^−1^)	6.31 ± 0.11

^1^ TPH: total petrol hydrocarbon; ^2^ LOI_550_: loss on ignition at 550 °C; ^3^ AHB: aerobic heterotrophic bacteria.

## Supplementary Materials

The following are available online at https://www.mdpi.com/1660-4601/17/11/4106/s1, Figure S1: Changes in the relative CO2 contents in liquid minimal medium supplemented with 1% (m v-1) fresh MK8 lubricant oil. Different letters in the same incubation time indicate statistical differences among treatments (*n* = 3, *p* ≤ 0.05), Figure S2: Bioconversion of total petrol hydrocarbons (TPH) in liquid minimal medium after 16 days. Different letters indicate statistical differences among treatments (*n* = 3, *p* ≤ 0.05).
