# Supplementary material for: Intensification of Ex Situ Bioremediation of Soils Polluted with Used Lubricant Oils: A Comparison of Biostimulation and Bioaugmentation with a Special Focus on the Type and Size of the Inoculum
Source: Int J Environ Res Public Health. 2020 Jun 9;17(11):4106. doi: 10.3390/ijerph17114106 (PMC7312492; doi:10.3390/ijerph17114106)
Supplement: Supplementary file 1 [file ijerph-17-04106-s001.pdf]

International Journal of
Environmental Research and Public Health

Supplementary Materials

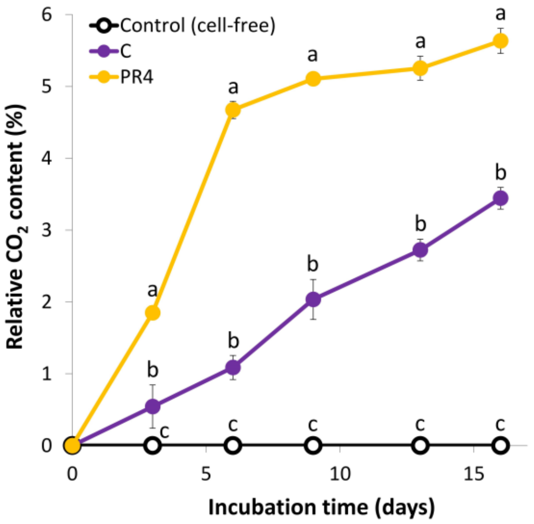

Figure S1. Changes in the relative CO_2 contents in liquid minimal medium supplemented with 1% (m v^{-1}) fresh MK8 lubricant oil. Different letters in the same incubation time indicate statistical differences among treatments (n=3, p≤0.05).

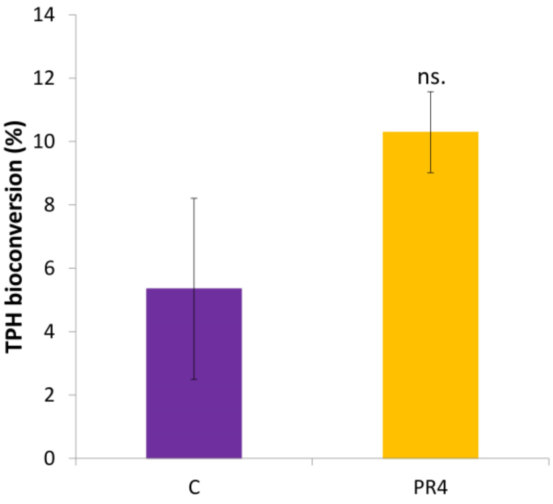

Figure S2. Bioconversion of total petrol hydrocarbons (TPH) in liquid minimal medium after 16 days. Different letters indicate statistical differences among treatments (n=3, p≤0.05).
